# Editorial Comment: Management of post TURP strictures

**DOI:** 10.1590/S1677-5538.IBJU.2020.01.05

**Published:** 2020-01-13

**Authors:** SB Kulkarni, O Joglekar, M Alkandari, PM Joshi, Luciano A. Favorito

**Affiliations:** 1 Kulkarni Reconstructive Urology Center, 3, Rajpath Society, Opp Vanaz Engineering, Paud Road, Pune, 411038, India; 1 Unidade de Pesquisa Urogenital - Universidade Estadual do Rio de Janeiro - Uerj, Rio de Janeiro, RJ, Brasil;; 2 Serviço de Urologia, Hospital Federal da Lagoa, Rio de Janeiro, RJ, Brasil

## COMMENT

Trans-urethral prostate resection (TURP) is one of the most common urologic surgeries and the urethral stricture is an important complication (1). In the present paper Dr. Kulkarni, in a prospective study with 170 patients shows that bulbar stricture is the most frequent affected area with stricture post TURP (143 patients) and that buccal mucosa graft (BMG) is safe, feasible and with long-term success in these patients that should be strongly considered. The overall success rate of Kulkarni with BMG was 82% in this paper. Other important presented result is that the Ventral approach is best suited for proximal bulbar strictures close to membranous urethra.

BMG placement can be ventral, dorsal or lateral (2–4). Ventral location provides the advantages of ease of exposure and good vascular supply by avoiding circumferential rotation of the urethra (5) and this paper shows that in post TURP stricture near the membranous urethra this technique is the best option. This is a very important publishing and we would like to congratulate the authors.
